# A Genomic Redefinition of *Pseudomonas avellanae* species

**DOI:** 10.1371/journal.pone.0075794

**Published:** 2013-09-25

**Authors:** Marco Scortichini, Simone Marcelletti, Patrizia Ferrante, Giuseppe Firrao

**Affiliations:** 1 Consiglio per la Ricerca e la Sperimentazione in Agricultura - Centro di Ricerca per la Frutticoltura, Roma, Italy; 2 Consiglio per la Ricerca e la Sperimentazione in Agricultura – Unità di Ricerca per la Frutticoltura, Caserta, Italy; 3 Dipartimento di Scienze Agrarie ed Ambientali, Università di Udine, Udine, Italy; University of the West of England, United Kingdom

## Abstract

The circumscription of bacterial species is a complex task. So far, DNA-DNA hybridization (DDH), 16S rRNA gene sequencing, and multiocus sequence typing analysis (MLSA) are currently the preferred techniques for their genetic determination. However, the average nucleotide identity (ANI) analysis of conserved and shared genes between two bacterial strains based on the pair-wise genome comparisons, with support of the tetranucleotide frequency correlation coefficients (TETRA) value, has recently been proposed as a reliable substitute for DDH. The species demarcation boundary has been set to a value of 95-96% of the ANI identity, with further confirmation through the assessment of the corresponding TETRA value. In this study, we performed a genome-wide MLSA of 14 phytopathogenic pseudomonads genomes, and assessed the ANI and TETRA values of 27 genomes, representing seven out of the nine genomospecies of *Pseudomonas* spp. sensu Gardan *et alii*, and their phylogenetic relationships using maximum likelihood and Bayesian approaches. The results demonstrate the existence of a well demarcated genomic cluster that includes strains classified as *P. avellanae, P. syringae* pv. *theae*, *P.* s. pv. *actinidiae* and one *P.* s. pv. *morsprunorum* strain all belonging to the single species *P. avellanae*. In addition, when compared with *P. avellanae*, five strains of *P.* s. pv. *tomato*, including the model strain DC3000, and one *P.* s. pv. *lachrymans* strain, appear as very closely related to *P. avellanae*, with ANI values of nearly 96% as confirmed by the TETRA analysis. Conversely, one representative strain, previously classified as *P. avellanae* and isolated in central Italy, is a genuine member of the *P. syringae* species complex and can be defined as *P.* s. pv. *avellanae*. Currently. The core and pan genomes of *P. avellanae* species consist of 3,995 and 5,410 putative protein-coding genes, respectively.

## Introduction

A rapid and destructive decline of cultivated hazelnut ( *Corylus* *avellana* L.) was first observed in northern Greece during the 1970s. Based on biochemical and nutritional tests, and a host range pathogenicity test, the bacterium responsible for the decline was named *Pseudomonas syringae* pv. *avellanae* and the disease was defined as bacterial canker of hazelnut [1]. The pathotype strain of the pathovar, namely BPIC631=NCPPB3487, was fully described and officially recognized some years later [2]. During the same period, a similar hazelnut disease was also observed in central Italy, and the causal agent was also identified as *P.* s. pv. *avellanae* [3]. Based on 16S rRNA gene sequence and fatty acid analyses, the causal agent of the bacterial canker of hazelnut in Greece and Italy was subsequently elevated to the species level and named *P. avellanae* [4]. A thorough DNA-DNA hybridization (DDH) study then confirmed *P. avellanae* as a distinct genomospecies, namely genomospecies 8, within the *P. syringae* species complex and some other phytopathogenic pseudomonads [5]. Genomospecies 8 also includes *P.* s. pv. *theae* [5], and *P.* s. pv. *actinidiae*, as subsequently pointed out [6,7]. However, molecular fingerprinting analyses using repetitive-sequence PCR [8] and classical tests such as the production of fluorescent pigments onto culture media [9] showed some clear differences between the *P. avellanae* populations found in Greece and Italy, and these differences were considered to be representative of the variability of the species. Therefore, two different lineages belonging to the same species were recognized and retained as originating separately [10] but evolving similarly to infect cultivated hazelnut trees [11].

An in-depth multilocus sequence typing (MLST) analysis (MLSA), based on fragments of the housekeeping genes *gapA*, *gltA*, *gyrB* and *rpoD*, and performed with many strains causing hazelnut bacterial canker isolated in Greece and Italy again placed all the previously classified *P. avellanae* strains into the *P. syringae* species complex. In particular, all the strains from Greece grouped into phylogroup 1, whereas the strains isolated in Italy were placed or into this phylogroup or into phylogroup 2 [11]. This reinstatement into the *P. syringae* species complex as pv. *avellanae* caused *bona fide* confusion with regard to naming the strains of the causal agent of hazelnut bacterial canker. In fact, although relevant taxonomic studies and/or reviews continue to confirm and treat *P. avellanae* as a distinct bacterial species [12,13,14,15], several other relevant studies aimed at comparing phytopathogenic bacteria and/or inferring evolutionary relationships among them, have followed the MLSA analysis of Wang et al. [11] and refer to two *P.* s. pv. *avellanae* phylogroups [16,17,18,19,20].

The circumscription of bacterial species is indeed a difficult task [21,22,23]. To date, DDH, 16S rRNA gene sequence analyses and comparison and MLSA analysis are the preferred techniques for genetically determining bacterial species. However, each of these techniques has some basic limitations including the impossibility of assembling cumulative databases based on DDH, the low variability and conservative nature of 16S rRNA genes not allowing sufficient resolution to infer clear taxonomic relationships, and putative bias in the selection of genes for the MLSA [24]. Recently, the average nucleotide identity (ANI) analysis of conserved and shared genes between two bacterial strains based on pair-wise genome comparisons [25], with support of the tetranucleotide frequency correlation coefficients (TETRA) value, has been proposed as a new standard for prokaryotic species definition [24] and is receiving wide acceptance. A genome assessment inferred using ANI well represented the degree of evolutionary distance between the compared genomes and an ANI value of 94% was proposed for replacing the classical DDH value of 70% for species demarcation [25]. A more extensive study largely confirmed the reliability of such an analysis and noted a slightly narrower boundary of 95% identity for the consistent substitution of the DDH value of 70% [26]. However, in confirming the robustness of the ANI analysis, Richter and Rosselló-Móra, set the species demarcation boundary at a value of 95-96% identity, and suggested further confirmation by the assessment of the TETRA value [24].

In this study, in addition to an MLSA based on seven housekeeping genes and maximum likelihood and Bayesian approaches, a genome wide phylogenetic analysis and consensus networks were performed with 14 genomes of phytopathogenic pseudomonads. Moreover, we analyzed the genome of 29 strains belonging to *Pseudomonas* spp. representing seven out of nine genomospecies sensu Gardan et al. [5] using the ANI analysis and the assessment of the TETRA values, for: a) clarifying the taxonomic relationships between the two *Pseudomonas* lineages associated with hazelnut bacterial canker in Greece and Italy (i.e., phylogroups 1 and 2), and b) to verify their genomic relationship within the genomospecies 8 and other genomospecies of phytopathogenic pseudomonads.

We revealed the existence of a well-demarcated *P. avellanae* species that also includes strains classified as *P. syringae* pv. *theae*, *P.* s. pv. *actinidiae* and one *P.* s. pv. *morsprunorum* strain. As the TETRA values confirmed the findings all such strains could putatively belong to *P. avellanae*. In addition, when compared to *P. avellanae*, five strains of *P.* s. pv. *tomato*, including the model strain DC3000, and one *P.* s. pv. *lachrymans* strain, showed ANI values very close to 96% which was confirmed by the TETRA analysis. Finally, one representative strain, previously classified as *P. avellanae* sensu Janse et al. [4] and isolated in central Italy, is, conversely, a genuine member of the *P. syringae* species complex and can be identified as *P.* s. pv. *avellanae*.

## Results

### Genome-wide sequence data and bacterial strains

We generated second generation sequence data from five *Pseudomonas* strains, namely *P. avellanae* BPIC631 (type-strain of the species), *P. avellanae* CRAFRUEC1, *P. syringae* pv. *theae* NCPPB2598 (type-strain of the pathovar) and *P.* s. pv. *syringae* CRAFRU11 and CRAFRU12 (isolated from *C* *. avellana* ). The genome size of the five strains was within the range of the previously sequenced and published *P. syringae* draft genomes (i.e., approximately 6 Mb). The main genomic features of the draft genomes are shown in Table 1. The sequences of the assemblies were deposited in NCBI GenBank under the following accession numbers: *P. avellanae* BPIC631=ATDK00000000; *P. avellanae* CRAFRUEC1=ATLL00000000; *P.* s. pv. *theae* NCPPB2598=ATDJ00000000; *P.* s. pv. *syringae* CRAFRU11=ATSU00000000 and *P.* s. pv. *syringae* CRFRU12=ATSV00000000. The bacterial strains and the respective accession numbers of their genomes utilized in this study are shown in Table 2.

**Table 1:** General features of draft genomes for the new sequenced *Pseudomonas* strains.

	PaveBPIC631	PaveCRAFRUec1	PthNCPPB2598	PsyCRAFRU11	PsyCRAFRU12
No. reads	5,823,418	13,639,825	11,607,142	3,381,518	3,350,210
No. contig	612	547	532	180	248
N50 (nt)	29,502	16,957	26,279	81.543	50.660
Average contig size (nt)	9,743	10,486	11,607	32.552	23.925
Total size (nt)	5,963,015	5,736,089	6,210,320	5,859,499	5,933,506
G+C content (%)	58.5	59.0	58.6	59.1	59.4
Calculated genome coverage	42	118	181	25	27

**Table 2:** Bacterial strain, strain code, genome accession number and genomospecies sensu Gardan et al. [5] regarding the *Pseudomonas* strains used in this study.

*Strain*	Strain code	Accession No.	Genomospecies
*Pseudomonas syringae pv. syringae*	PsyB728a	NC_007005	1
*Pseudomonas syringae pv. syringae*	PsyCRAFRU11	ATSU00000000	1
*Pseudomonas syringae pv. syringae*	PsyCRAFRU12	ATSV00000000	1
*Pseudomonas syringae pv. aceris*	PsacM302273	AEA000000000	1
*Pseudomonas syringae pv. pisi*	Ppi1704B	AEAI00000000	1
*Pseudomonas syringae pv. avellanae*	PsaveCRAPAV013	AKCJ00000000	1
*Pseudomonas syringae pv. aesculi*	Psae2250	ACXT00000000	2
*Pseudomonas syringae pv. glycinea*	PgyRace4	ADWY00000000	2
*Pseudomonas syringae pv. mori*	Pmo301020	AEAG00000000	2
*Pseudomonas syringae pv. phaseolicola*	Pph1448A	NC_005773, NC_007274, NC_007275	2
* Pseudomonas savastanoi *	PsvNCPPB3335	ADMI02000000	2
*Pseudomonas syringae pv. lachrymans*	PlaM302278	AEAM00000000	2
*Pseudomonas syringae pv. morsprunorum*	PmpM302280	AEAE00000000	3
*Pseudomonas syringae pv. tomato*	PtoDC3000	NC_004578, NC_004632, NC_004633	3
*Pseudomonas syringae pv. tomato*	PtoT1	ABSM00000000	3
*Pseudomonas syringae pv. tomato*	PtoMax13	ADFZ00000000	3
*Pseudomonas syringae pv. tomato*	PtoTK40	ADFY00000000	3
*Pseudomonas syringae pv. tomato*	PtoNCPPB1108	ADGA00000000	3
*Pseudomonas syringae pv. oryzae*	Por1_6	ABZR00000000	4
* Pseudomonas viridiflava *	PvirUASWS0038	AMQP00000000	6
*Pseudomonas avellanae*	PaveBPIC631	ATDK00000000	8
*Pseudomonas avellanae*	PaveCRAFRUec1	ATLL00000000	8
*Pseudomonas syringae pv. actinidiae*	PanNCPPB3739	AFTH00000000	8
*Pseudomonas syringae pv. actinidiae*	PanNCPPB3871	AFTF00000000	8
*Pseudomonas syringae pv. actinidiae*	PanCRAFRU8.43	AFTG00000000	8
*Pseudomonas syringae pv. actinidiae*	PanM302091	AEAL00000000	8
*Pseudomonas syringae* pv. *theae*	PthNCPPB2598	ATDJ00000000	8
* Pseudomonas cannabina pv. alisalensis*	PcalBS91	Taxon ID 2516653056*	9
*Pseudomonas fluorescens*	PfA506	NC_017911	
* Pseudomonas putida *	PpuUW4	NC_019670	

### Phylogeny based on MLSA

**Figure 1:**
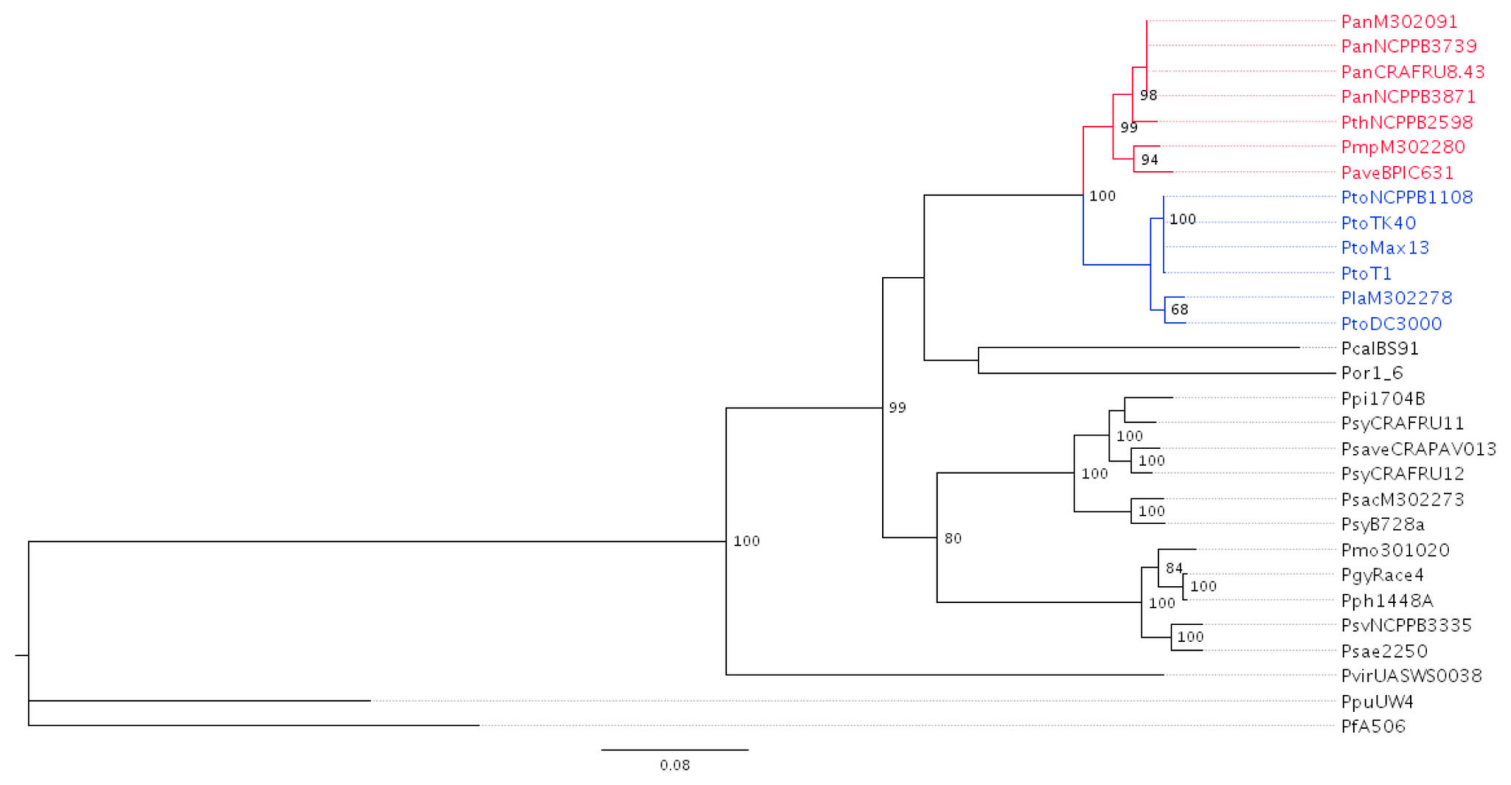
Phylogenetic relationships among representative strains of phytopathogenic *Pseudomonas* species and *P. syringae* pathovars. The phylogenetic tree was constructed using 6,579 concatenated nucleotides of seven housekeeping genes (*argS, dnaQ, gltA, gyrB, recA, rpoB* and *rpoD*) with bootstrap values greater than 65 per cent (1,000 replicates) shown at the nodes. The phylogenetic relationships were inferred using the maximum likelihood (ML) method and the GTR + I + G as the best model with the PHYLIP package. Strain members of genomospecies 8 (*P. avellanae*) sensu Gardan et al. [5], including also *P*. *s*. pv. *morsprunorum* M302280, are shown in red, whereas strain members of genomospecies 2 (P. *s*. pv. *lachrymans* M302278) and 3 (P. *s*. pv. *tomato*) are in blue. *P. fluorescens* A506 and *P* *. putida* UW4 were included as outgroups.

**Figure 2:**
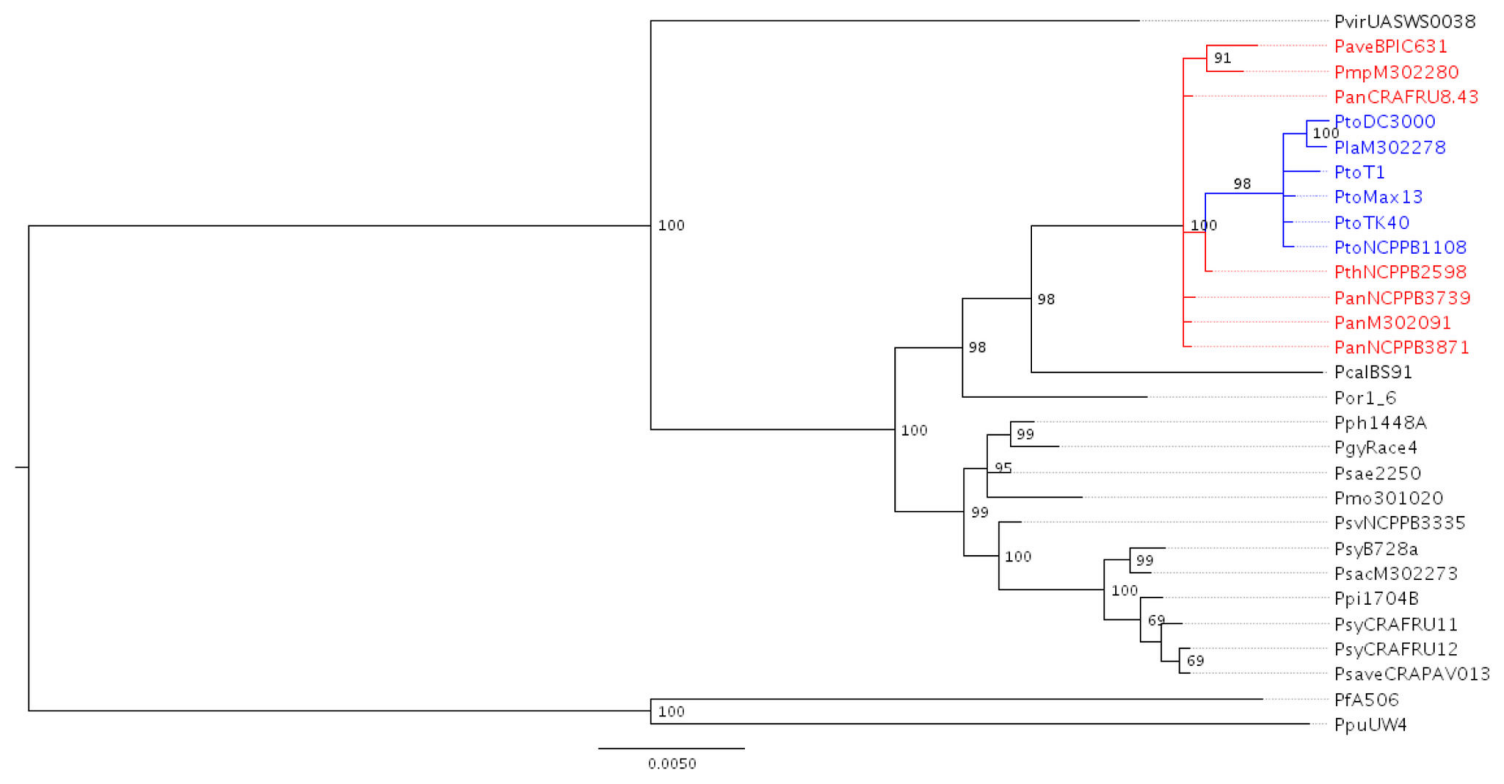
Bayesian phylogenetic tree showing relationships among representative strains of phytopathogenic *Pseudomonas* species and *P. syringae* pathovars. The phylogenetic tree was constructed using 2,193 concatenated amino acid of seven housekeeping genes (*argS, dnaQ, gltA, gyrB, recA, rpoB* and *rpoD*) with bootstrap values (100,000 generations) shown at the nodes. Strain members of genomospecies 8 (*P. avellanae*) sensu Gardan et al. [5], including also *P*. *s*. pv. *morsprunorum* M302280, are shown in red, whereas strain members of genomospecies 2 (P. *s*. pv. *lachrymans* M302278) and 3 (P. *s*. pv. *tomato*) are in blue. *P. fluorescens* A506 and *P* *. putida* UW4 were included as outgroups. To note that strains of genomospecies 2 and 3 are embedded into strains of genomospecies 8. The interior node values of the tree are clade credibility values based on the posterior credibility values produced by MrBayes.

A ML and a Bayesian phylogenetic trees of the concatenated DNA sequences of seven housekeeping genes, namely *argS, dnaQ, gltA, gyrB, recA, rpoB* and *rpoD* (a total of 6,579 nucleotides) of 27 phytopathogenic pseudomonads representative of seven genomospecies sensu Gardan et al. [5] and two *Pseudomonas* spp. used as outgroups are shown in Figure 1 and Figure S1. Both trees revealed the presence of a cluster composed by two well-defined subclusters, including *Pseudomonas* members of genomospecies 2 and 3 (*P. s*. pv. *lachrymans* and *P. s.* pv. *tomato*, respectively) and 8 (*P. avellanae*, *P. s.* pv. *theae*, and *P. s.* pv. *actinidiae*) in addition to *P.* s. pv. *morsprunorum* M302280 putatively belonging to genomospecies 3. The significance of such clustering is very high in both trees. The strains representative of the species *P.* s. pv. *oryzae*, *P* *. viridiflava* and *P* *. cannabina* pv. *alisalensis* resulted well distinct from the above described clusters (Figure 1 and Figure S1). The corresponding phylogenetic trees constructed using amino acid alignment and ML and Bayesian approaches after alignment with a hidden Markov model (i.e., a concatenation of 2,193 amino acid sequences) were substantially similar to those based on DNA sequences (Figure 2, Figure S2). In this case, both trees also significantly revealed very close relationships between the strains of genomospecies 3 and 8 and the location of *P.* s. pv. *morsprunorum* M302280 within genomospecies 8. Remarkably, the Bayesian tree also showed *P.* s. pv. *tomato* and *P.* s. pv. *lachrymans* embedded within the strains of genomospecies 8 (Figure 2).

**Figure 3:**
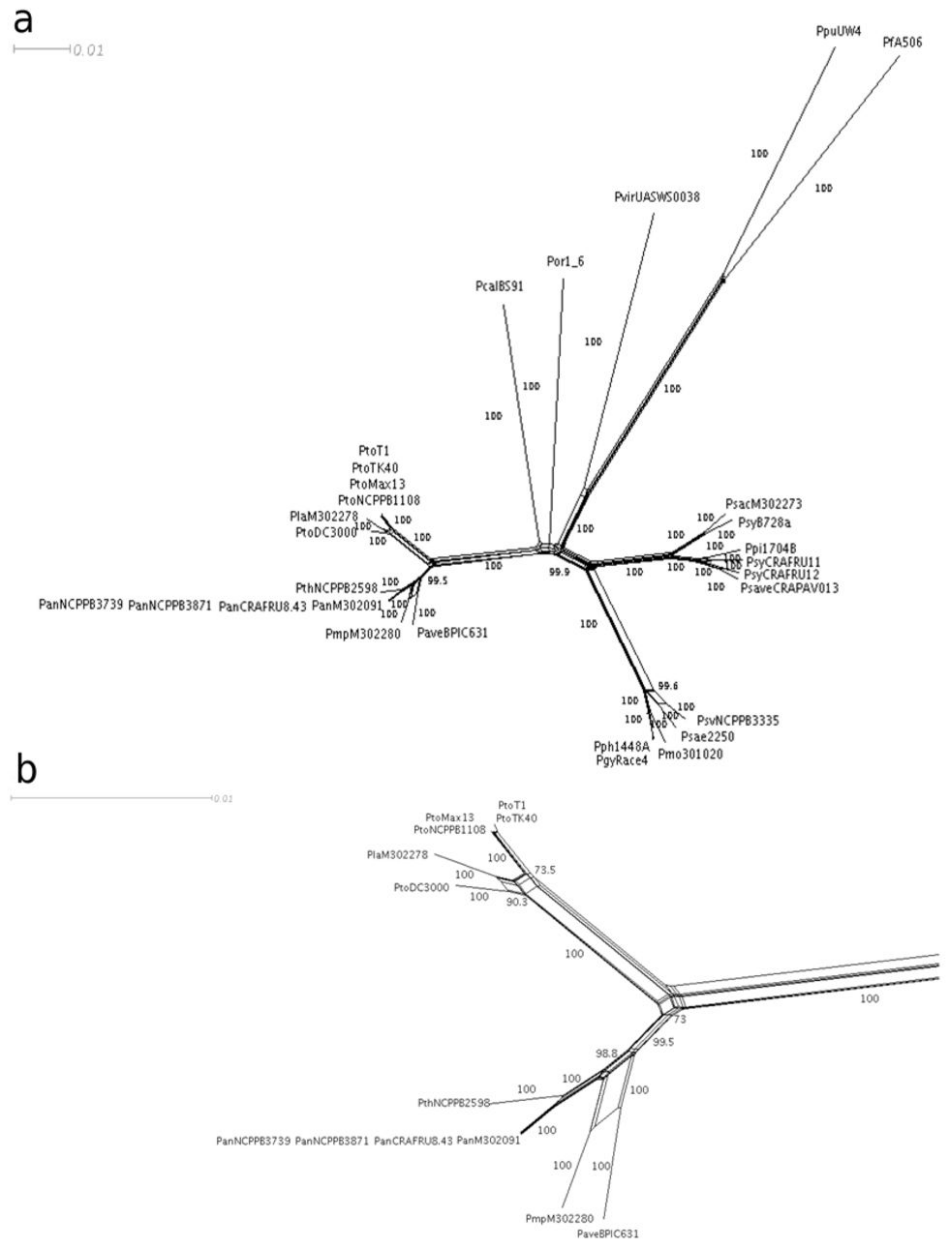
Split network of the concatenated 6,579 nucleotides for 29 *Pseudomonas* spp. genomes. A) Strains of genomospecies 3 (P. *s*. pv. *tomato*) and 2 (P. *s*. pv. *lachrymans*) and genomospecies 8 (*P. avellanae*, P. *s*. pv. *actinidiae, P*. *s*. pv. *theae*) sensu Gardan et al. [5] as well as *P*. *s*. pv. *morsprunorum* M30228, clustered apart from the other representative strains of genomospecies 1, 4, 6 and 9. Bootstraps values higher than 65% are shown. B) Particular of the split network of Figure 5A regarding strains of *Pseudomonas avellanae* species and the closely-related *P. syringae* pv. *tomato* and *P*. *s*. pv. *lachrymans* strains. Bootstraps values higher than 65% are shown.

The split network based on the concatenated alignment of 6,579 nucleotides provided a significant and very similar phylogenetic analysis when compared to the PhyML and Bayesian trees (Figure 3a and 3b). In fact, the strains of genomospecies 3 and 8 and *P.* s. pv. *morsprunorum* M302280 clustered together albeit into two separate subclusters. In addition, the strains of genomospecies 1 and 2 clustered separately, with *P.* s. pv. *oryzae*, *P* *. viridiflava* and P. *c*. pv. *cannabina* again appearing as separate *taxa* from the other genomospecies.

### Genome-wide phylogeny

**Figure 4:**
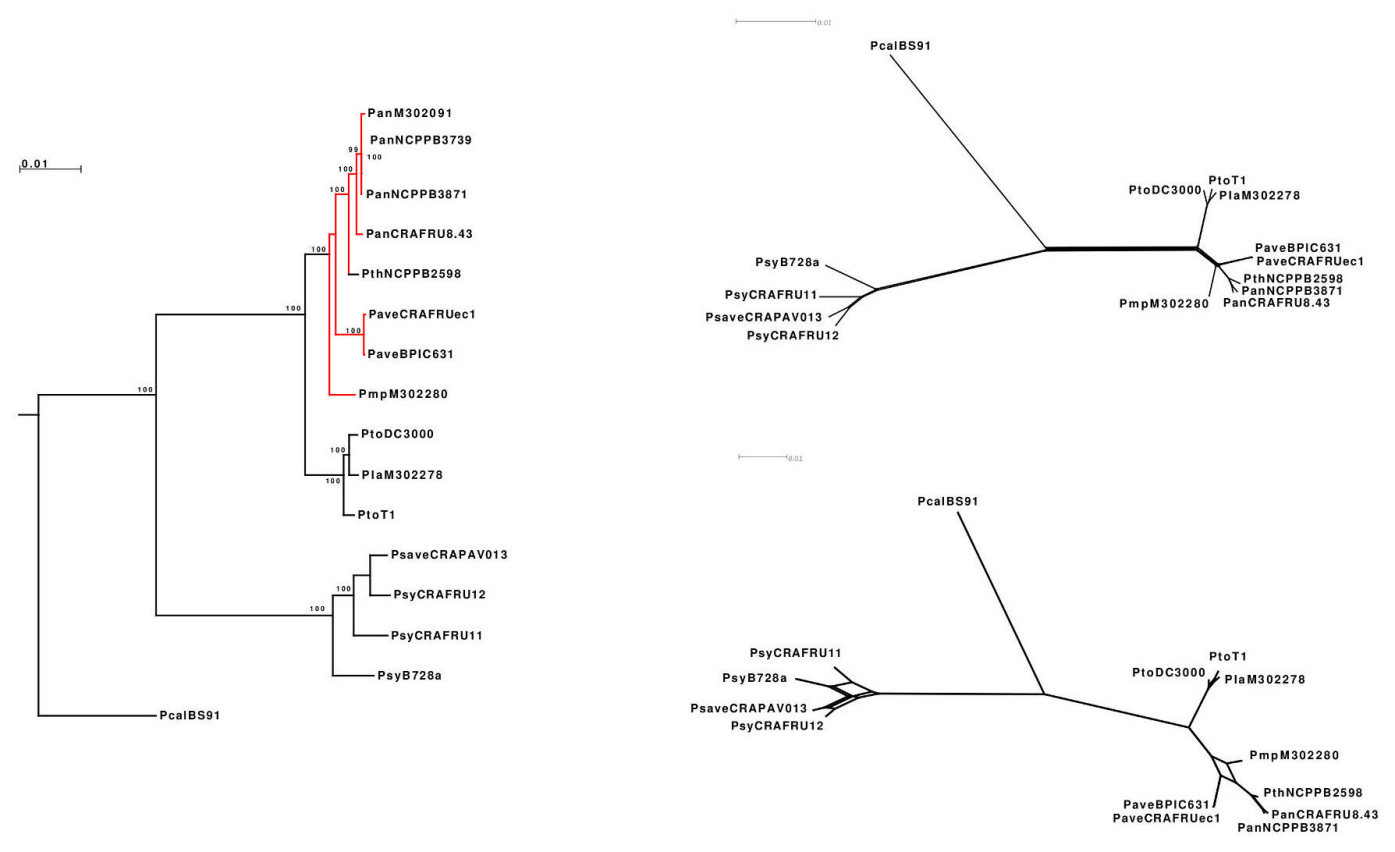
Genome wide phylogeny of 14 phytopathogenic pseudomonads inferred with concatenated protein sequences from 1,920 genes for a total of 612,057 amino acid sites. a) Maximum likelihood tree showing the phylogenetic relationships between strain of *P. avellanae* species (in red) with strains of *P*. *s*. pv. *tomato*/*P*. *s*. pv. *lachrymans*, *P*. *s*. pv. *syringae*/*P*. *s*. pv. *avellanae* and *P* *. cannabina* pv. *alisalensis*; b) split network tree showing a tree-like structure indicating the absence of systematic error in the definition of strain clusters; c) consensus network showing the strictly connected evolutionary history of strains of genomospecies 3 (P. *s*. pv. *tomato* and *P*. *s*. pv. *morsprunorum*) and 8 (*P. avellanae*) sensu Gardan et al. [5].

The ML tree of the concatenated protein sequences of 1,920 genes (for a total of 612,057 amino acids) in 14 phytopathogenic pseudomonads representative of five genomospecies sensu Gardan et al. [5] is shown in Figure 4a. The tree revealed the presence of well-defined clusters containing: a) the members of genomospecies 1 (*P. s*. pv. *syringae* and *P. s*. pv. *avellanae* CRAPAV 013); b) members of genomospecies 2 (*P. s*. pv. *lachrymans*) and 3 (*P. s*. pv. *tomato*); and c) members of genomospecies 3 ( *P. s. morsprunorum* ) and 8 (*P. avellanae*, *P. s*. pv. *theae* and *P. s*. pv. a*ctinidiae*). *P* *. cannabina* pv. *alisalensis* BS91 (genomospecies 9) clustered separately. The relatively long distances between the clusters and the high bootstrap values support the notion that the above-described clusters represent distinct evolutionary lineages.

This concatenation includes a large fraction of the genome thus providing sequences that are long enough to overcome sampling error. Nonetheless, the robustness of the phylogenetic reconstruction might not be easily evaluated even with 100% non parametric bootstrap support [27], as the latter is a statistical technique designed to prevent sampling error and not systematic error [28]. Consequently, an assessment of the robustness of the phylogenetic reconstruction was obtained from the split network shown in Figure 4b, which reveals that the partition of the strains into clusters is substantially tree-like, thus indicating the absence of systematic error in the definition of the strain clusters.

With reference to the strain composition of the individual clusters, we observed a limited, yet detectable presence of contradictory phylogenetic signals associated with complex (i.e., nonbifurcating) evolutionary histories of the individual strains. The lack of full congruence of the individual gene phylogenies, likely due to the role that horizontal DNA transfer from other relatives had in shaping the evolution of the individual strains, was additionally highlighted by a consensus network. Figure 4c shows the consensus network obtained with a cutoff of 0.1 (i.e., showing the edges that occur in a proportion of the gene trees higher than 10%). Therefore, in more than 10% (i.e., 192 trees) cases, the branching order among *P.* s. pv. *morsprunorum*, *P. avellanae* and *P.* s. pv. *actinidiae*/*P.* s. pv. *theae* is different from that displayed in the ML tree of Figure 4a, highlighting the strictly connected and ongoing evolutionary history of the strains of genomospecies 3 and 8.

### Average nucleotide identity (ANI) and tetranucleotide frequency correlation coefficients (TETRA) analysis

**Figure 5:**
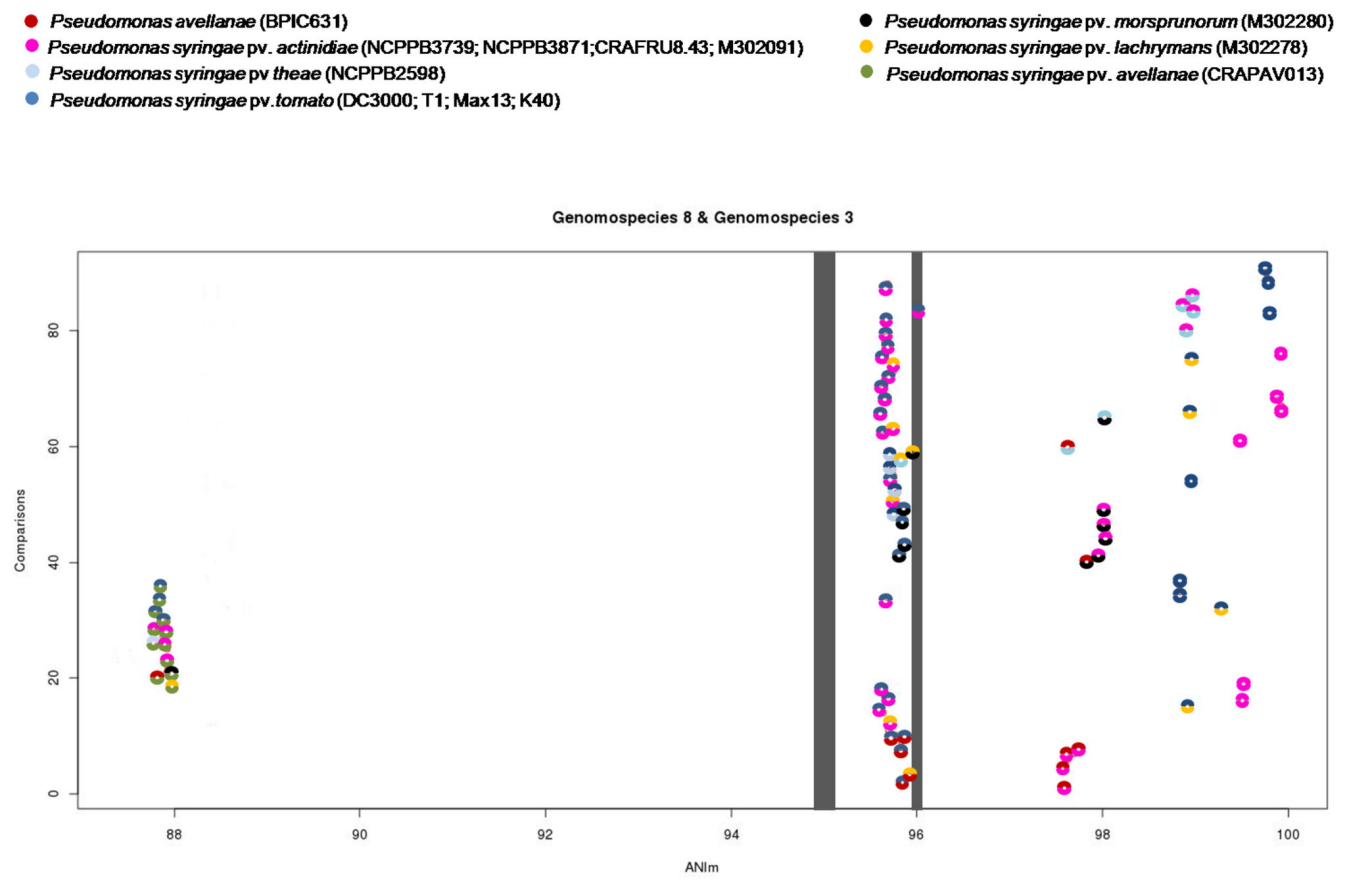
Graphical representation of the average nucleotide identity (ANI) comparison between genomes of *Pseudomonas* strains.

The five newly sequenced genomes, together with those of another 22 phytopathogenic *Pseudomonas* spp. representative of seven of the nine genomospecies described by Gardan et al. after DDH assessment [5], and two *Pseudomonas* spp., namely *P. fluorescens* A506 and *P* *. putida* UW4 used as outgroups, were cross-compared to reveal their total similarities in terms of the amount of sequence identity. The ANI value calculations, based on the MUMmer alignment of each sequence pair, are reported in Table 3 and Table S1. A graphical representation of the analysis obtained using R statistic-based software is shown in Figure 5. ANI analysis has recently been proposed as a new standard for inferring robust taxonomic relationships between bacterial species based on genome comparison and it has been assumed that values of 95% or 95-96% for ANI correspond to the 70% of the DDH reassociation value for demarcating bacterial species.

**Table 3:** Average nucleotide identity (ANI) values calculated between genomes of 13 *Pseudomonas* strains belonging to genomospecies 3 and 8 sensu Gardan et al. [5].

	PaveBPIC631	PthNCPPB2598	PanNCPPB3739	PanNCPPB3871	PanCRAFRU8.43	PanM302091	PmpM302280	PlaM302278	PtoDC3000	PtoK40	PtoMax13	PtoT1	PtoNCPPB1108
PaveBPIC631	—	**97,59**	**97,63**	**97,62**	**97,60**	**97,70**	**97,83**	**95,92**	**95,86**	**95,83**	**95,84**	**95,79**	**95,78**
PthNCPPB2598	**97,61**	—	**98,94**	**98,94**	**98,91**	**98,96**	**98,06**	**95,80**	**95,75**	**95,74**	**95,74**	**95,73**	**95,72**
PanNCPPB3739	**97,67**	**98,95**	—	**99,92**	**99,51**	**99,92**	**98,04**	**95,78**	**95,69**	**95,68**	**95,66**	**95,61**	**95,69**
PanNCPPB3871	**97,64**	**98,92**	**99,89**	—	**99,46**	**99,91**	**98,02**	**95,77**	**95,66**	**95,66**	**95,64**	**95,60**	**95,66**
PanCRAFRU8.43	**97,60**	**98,91**	**99,49**	**99,48**	—	**99,52**	**97,98**	**95,73**	**95,72**	**95,67**	**95,65**	**95,64**	**95,64**
PanM302091	**97,71**	**98,96**	**99,89**	**99,89**	**99,52**	—	**98,04**	**95,75**	**95,72**	**95,68**	**95,66**	**95,66**	**95,68**
PmpM302280	**97,83**	**98,05**	**98,02**	**98,02**	**97,96**	**98,04**	—	**95,87**	**95,84**	**95,84**	**95,86**	**95,80**	**95,78**
PlaM302278	**95,91**	**95,79**	**95,77**	**95,76**	**95,72**	**95,75**	**95,87**	—	**99,28**	**98,91**	**98,93**	**98,91**	**98,97**
PtoDC3000	**95,86**	**95,76**	**95,68**	**95,66**	**95,70**	**95,71**	**95,84**	**99,29**	—	**98,85**	**98,85**	**98,83**	**98,91**
PtoK40	**95,83**	**95,75**	**95,67**	**95,65**	**95,68**	**95,68**	**95,85**	**98,91**	**98,83**	—	**99,81**	**99,75**	**99,77**
PtoMax13	**95,84**	**95,74**	**95,66**	**95,64**	**95,64**	**95,66**	**95,86**	**98,93**	**98,83**	**99,80**	—	**99,74**	**99,76**
PtoT1	**95,80**	**95,73**	**95,62**	**95,61**	**95,65**	**95,66**	**95,82**	**98,94**	**98,86**	**99,75**	**99,79**	—	**99,73**
PtoNCPPB1108	**95,78**	**95,73**	**95,68**	**95,66**	**95,64**	**95,68**	**95,79**	**98,96**	**98,90**	**99,76**	**99,76**	**99,73**	—

To note that strains of genomospecies 8, including also *P.* s. pv. *morsprunorum* M302280, showed ANI values higher than 97,5%, whereas strains of genomospecies 3 (*P. s*. pv. *tomato* and *P. s*. pv. *lachrymans*) showed ANI values close to 96%.

 The figure shows the ANI analysis values of *Pseudomonas avellanae* species (*P. avellanae*, *P. s*. pv. *actinidiae*, *P. s*. pv. *theae* and *P. s*. pv. *morsprunorum* M302280), with *P. s*. pv. *tomato* (strains DC3000, T1, Max13 and K40), *P. s*. pv. *lachrymans* (M302278) and *P. s*. pv. *avellanae* (CRAPAV013).

We found that the strains of *P. avellanae*, *P.* s. pv. *theae*, and *P.* s. pv. *actinidiae* (genomospecies 8) displayed ANI values that were consistently higher than 97.5% in any reciprocal comparison. The four *P.* s. pv. *actinidiae* strains showed ANI values that were always higher than 99% in intrapathovar comparison. Remarkably, *P.* s. pv. *morsprunorum* M302280 showed ANI values that were consistently higher than 97.8% when compared to the strains of genomospecies 8. In addition, *P.* s. pv. *tomato* strains DC3000, NCPPB1108, T1, Max13 and K40 and *P.* s. pv. *lachrymans* M302278 showed ANI values comprised between 95 and 96% in any reciprocal comparison with strains of genomospecies 8 and *P.* s. pv. *morsprunorum* M302280 (Table 3 and Figure 5). Conversely, the *Pseudomonas* strain CRAPAV013, which was previously known as ISPaVe013, isolated in central Italy in 1992 from a hazelnut tree showing bacterial canker disease, and initially identified as *P. syringae* pv. *avellanae* [3], yet later reclassified as *P. avellanae* [4], showed ANI values lower than 88% when compared to the strains of genomospecies 8. In contrast, CRAPAV013 showed ANI values higher than 95.5% when compared to the *P. syringae* strains of genomospecies 1 (Table S1). The ANI analysis revealed also values higher than 97.7% in any reciprocal comparison for the strains of genomospecies 2, whereas the ANI values ranged from 95.3% (*P. s*. pv. *aceris* M302273 versus *P. s*. pv. *pisi* 1704B) to 98.6% (*P. s*. pv. *aceris* M302273 versus *P. s*. pv. *syringae* B728a) for strains belonging to genomospecies 1. The strains representative of genomospecies 4, 6, and 9, namely *P.* s. pv. *oryzae* 1.6, *P* *. viridiflava* UASWS0038 and P. *c*. pv. *alisalensis* BS91, respectively and the outgroups *P. fluorescens* A506 and *P* *. putida* UW4 showed ANI values lower than 88.2% when reciprocally compared to the other genomospecies strains (Table S1). Concerning the close relationships found for the strains of genomospecies 3 and 8, the TETRA analysis confirmed the results obtained with the ANI analysis, with values that were always higher than 0.997 in any reciprocal comparison, (Table 4). This analysis was intended to verify whether an alignment-free genomic feature can be used to circumscribe bacterial species [24].

**Table 4:** Tetranucleotide frequency correlation coefficients (TETRA) values calculated for 13 *Pseudomonas* **strains genomes belonging to genomospecies 3 and 8 sensu Gardan et al. [5], and including also P. s**. **pv. morsprunorum M302280.**

PaveBPIC631	**0,99831**	**0,99802**	**0,99836**	**0,99846**	**0,99826**	**0,99855**	**0,99805**	**0,99745**	**0,99802**	**0,99804**	**0,99789**	**0,99791**
	PthNCPPB2598	**0,99930**	**0,99946**	**0,99908**	**0,99954**	**0,99929**	**0,99911**	**0,99844**	**0,99921**	**0,99932**	**0,99935**	**0,99920**
		PanNCPPB3739	**0,99987**	**0,99918**	**0,99957**	**0,99902**	**0,99883**	**0,99801**	**0,99881**	**0,99888**	**0,99894**	**0,99889**
			PanNCPPB3871	**0,99918**	**0,99964**	**0,99914**	**0,99886**	**0,99825**	**0,99884**	**0,99893**	**0,99899**	**0,99893**
				PanCRAFRU8.43	**0,99902**	**0,99899**	**0,99863**	**0,99793**	**0,99870**	**0,99859**	**0,99851**	**0,99857**
					PanM302091	**0,99947**	**0,99938**	**0,99828**	**0,99928**	**0,99937**	**0,99927**	**0,99942**
						PmpM302280	**0,99935**	**0,99804**	**0,99908**	**0,99912**	**0,99891**	**0,99908**
							PlaM302278	**0,99872**	**0,99968**	**0,99968**	**0,99947**	**0,99966**
								PtoDC3000	**0,99870**	**0,99861**	**0,99878**	**0,99864**
									PtoK40	**0,99995**	**0,99983**	**0,99988**
										PtoMax13	**0,99988**	**0,99988**
											PtoT1	**0,99979**
												PtoNCPPB1108

The analysis confirmed the ANI value assessment by showing TETRA values always higher than 0,99. The analysis confirmed the ANI value assessment by showing TETRA values always higher than 0,99.

### The core and the pan genome of *Pseudomonas avellanae* species

**Figure 6:**
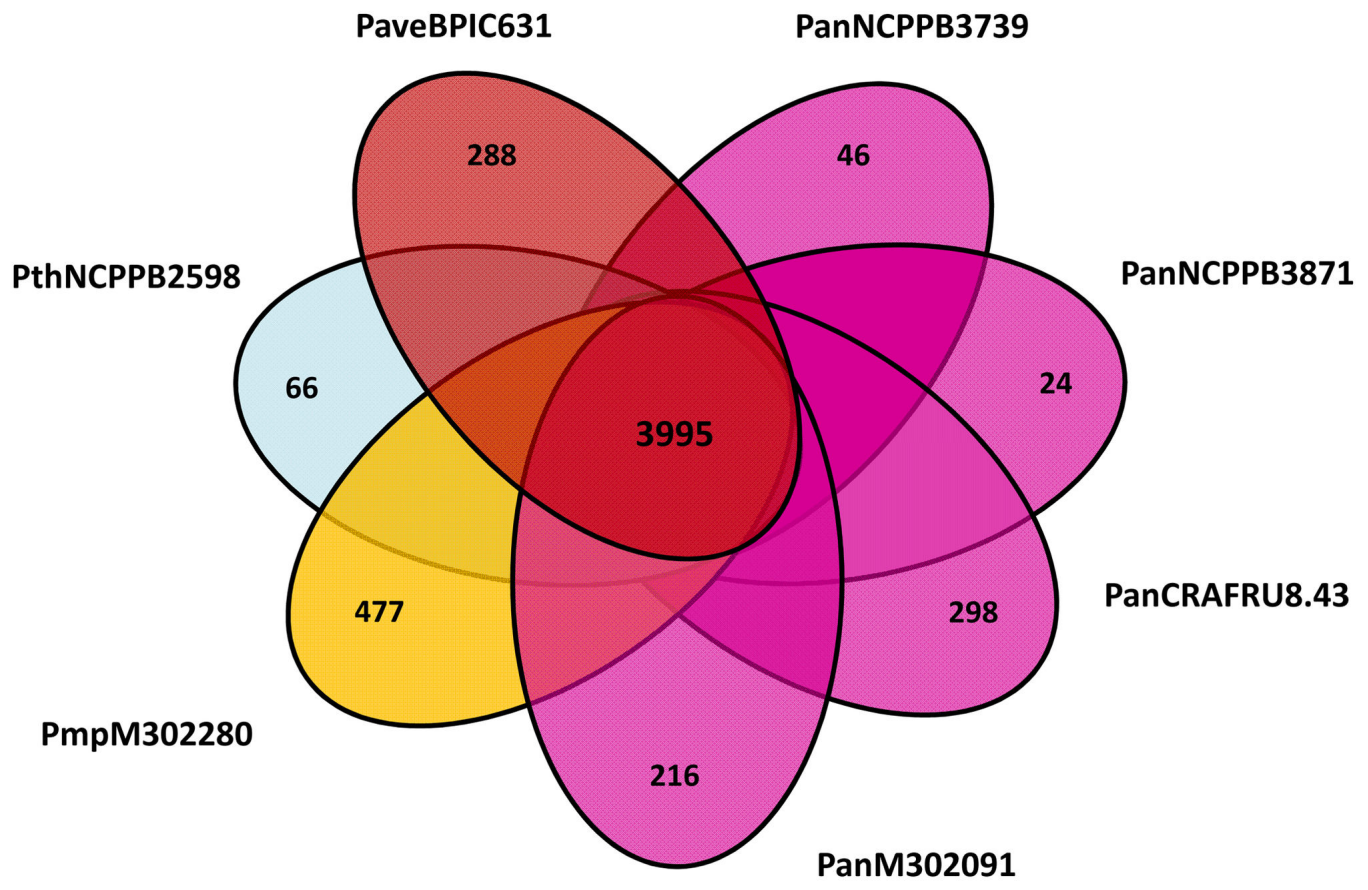
The core genome of *Pseudomonas avellanae* species. Each strain is represented by an oval that is colored according to the current and traditional strain determination. The number of orthologous coding sequences (CDSs) shared by all strains (i.e., the core genome) is in the center (i.e., 3,995). Numbers in the non-overlapping portions of each oval show the number of CDSs unique to each strain.

**Figure 7:**
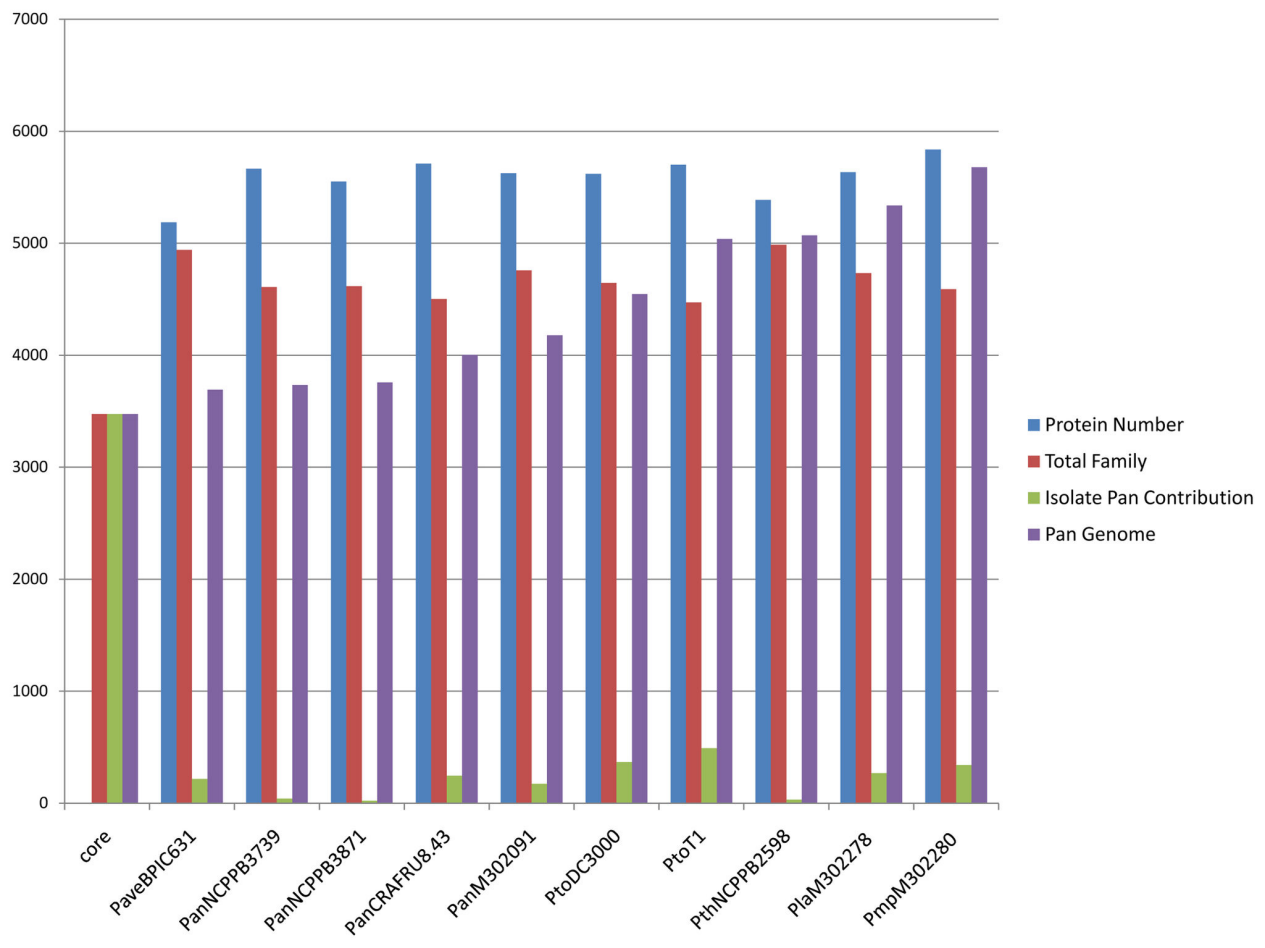
The pan genome of *Pseudomonas avellanae* species. The *P. avellanae* pan genome consists of 5,410 ORFs. The graphic shows also the total putative proteins and protein family number found for each strain.

The core genome consists of the number of genes found in all the sequenced strain genomes of a species, whereas the pan genome comprises the sum of the core genome and genes of the “flexible” genome (i.e., unique genes that are present in different strains of the species and are typically acquired through lateral gene transfer) [29,30]. We applied an analysis solely to the strains which that showed ANI values clearly higher than 96%. We defined the core genome for the seven strains of the species *P. avellanae*, as defined here by phylogenetic and non-phylogenetic genome analyses, as 3,995 putative protein-coding genes (Figure 6). Each strain exhibited a relatively small set of unique genes comprising the pan genome of the species with 5,410 ORFs (Figure 7). The overall percentage of strain-specific genes varied considerably ranging from 0.6% (*P. s*. pv. *actinidiae* NCPPB3871) to 11.9% (*P. s*. pv. *morsprunorum* 302280). The pan genome of the species, based on the seven strains here assessed, would seem similar, in terms of CDSs number, to that of the *P. syringae* species complex which was inferred with 19 strains infecting a vast array of plant species and consisting of 12,829 CDSs [31].

### The type III secretion system effector proteins

**Figure 8:**
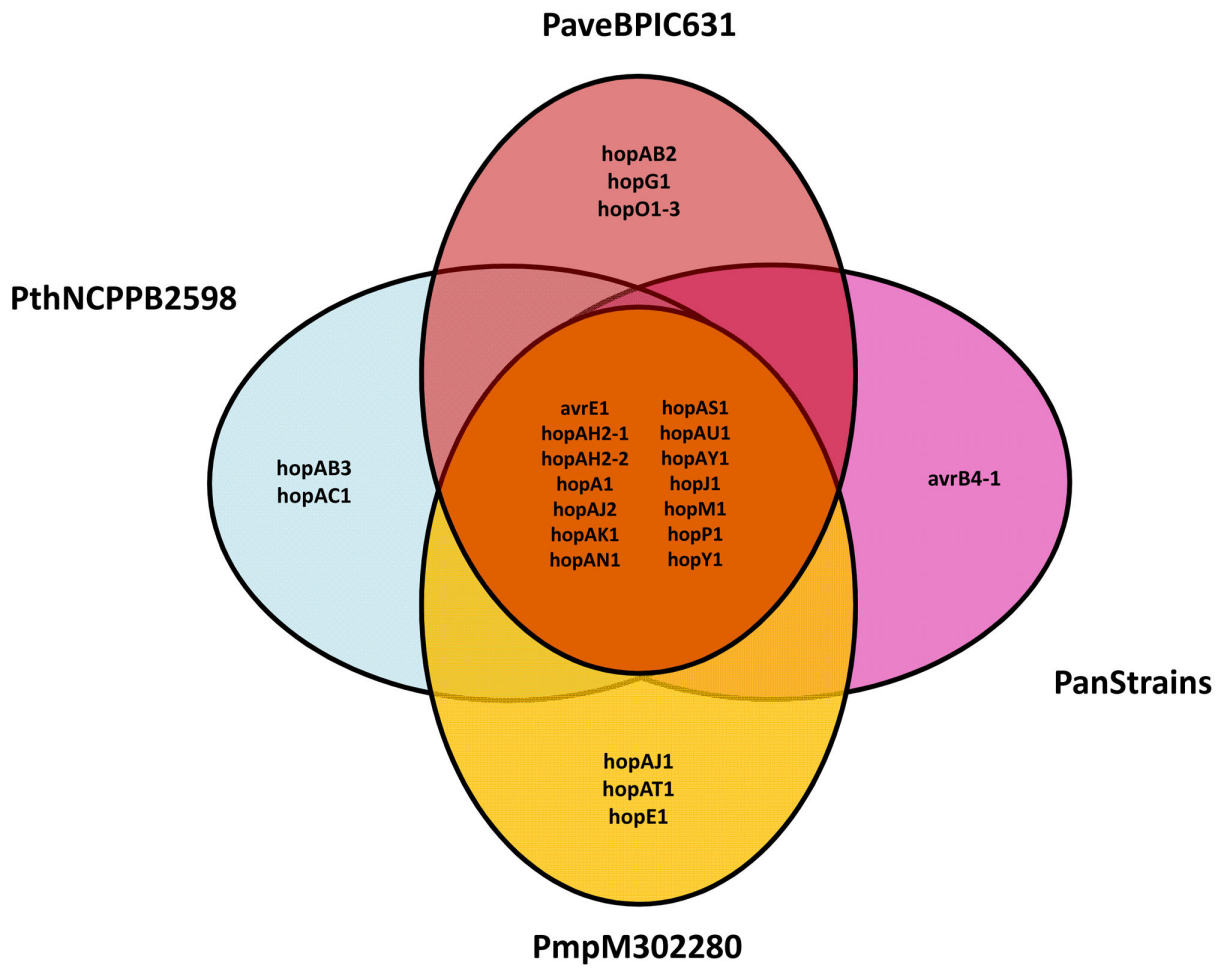
Venn diagram of the type III effector gene complements of *Pseudomonas avellanae* species. The diagram is based on the comparison of the same complement of other sequenced plant pathogenic pseudomonads and showing full identity in the reciprocal comparison. The effector proteins conserved among the seven strains are indicated in the centre of the diagram.

A comparison of the effector repertoires of the seven strains of *P. avellanae* species based on the complete dataset of effector proteins identified in 19 *P. syringae* as assessed by Baltrus et al. [31], revealed a core set of 14 putative effector genes that are conserved in all strains (Figure 8). In addition, each pathovar belonging to the species complex showed a unique set of effector proteins. The effector *hopM1*, present in all seven strains of *P. avellanae*, provides evidence for the pathogenic differentiation of the strains of the *P. avellanae* species complex from *P.* s. pv. *tomato* and *P.* s. pv. *lachrymans*. In fact, Baltrus et al. [31] found a recombination event at the *hopM1* locus that split the strains of clade I (*P. s*. pv. *actinidiae, P. s*. pv. *morsprunorum, P. s*. pv. *tomato* and *P. s*. pv. *lachrymans*) into two clearly separated groups: *P.* s. pv. *actinidiae* M302091-*P.* s. pv. *morsprunorum* M302280 and *P.* s. pv. *tomato* DC3000-*P.* s. pv. *lachrymans* 106.

## Discussion

The technical and conceptual definition of bacterial species continues to be uncertain representing a challenging task [32,33]. Currently, a prokaryotic species is defined as “a category that circumscribes a genomically coherent groups of individual strains sharing a high degree of similarity in independent features, comparatively tested under highly standardized conditions” [34]. Whole-genome DDH has traditionally been considered the gold standard in bacterial taxonomy, with a bacterial species (genomospecies) including strains with approximately 70% or greater DNA-DNA relatedness and with 5°C or less ΔT_m_ [35]. However, due to its high cost, low reproducibility and, mainly, for the impossibility of generating cumulative databases, DDH has been largely replaced by 16S rRNA gene sequencing and comparison even though the latter technique is not deemed a suitable candidate to fully replace DDH [36]. A technical definition indicates that a prokaryotic species is considered as “a group of strains characterized by a certain degree of phenotypic consistency and showing 70% of DNA-DNA binding and over 97% of 16S rRNA gene-sequence identity” [23]. Within phytopathogenic pseudomonads, Gardan et al. [5], by performing an extensive DDH assessment of many *P. syringae* pathovars, delineated a robust classification based on nine discrete genomospecies. However, according to the international taxonomic rules, a genomospecies should be named formally only when phenotypic characters are available to differentiate them [35]. Since Gardan et al. [5] did not find out reliable phenotypic traits (i.e., carbon source assimilation) to clearly distinguish all nine genomospecies, they could officially describe only two species (i.e., *Pseudomonas* *cannabina* , *P* *. tremae* ). Consequently, most of such genomospecies still have to formally be described.

However, several studies have shown that the sequencing of a single, highly conserved gene, such as the 16S rRNA gene, fails to intercept the true genome-wide divergence between two strains [24,37,38]. More recently, the analysis of several (i.e., four to seven) housekeeping genes or of their fragments, MLSA, was recommended as the primary approach for substituting DDH [22,23]. This technique is now widely used for inferring phylogenetic relationships among bacterial strains and/or single genes in their genomes. However, from a strict taxonomic standpoint, MLSA has some limitation mainly represented by putative bias in the gene selection for the analysis [24].

The advent of genomic has begun to provide complete or draft bacterial genomes that can easily be retrieved from public databases. Based on the opportunity offered by the availability of large gene datasets, MLSA could be extended to a relevant part of the genome, minimizing the gene selection bias and sampling error [39,40]. In the search of criteria to consistently replace DDH and the other techniques by exploiting genomic data, average nucleotide identity (ANI) analysis has been proposed [25,26,41] and validated for the reliable demarcation of bacterial species using a non-phylogenetic approach [24,42]. The boundary for species delineation inferred via ANI is currently set between 95 and 96%, which corresponds to the 70% of the DDH analysis, though a group-specific finer calibration appears to be desirable [24]. In this work, we performed a study to investigate the taxonomic relationships among some phytopathogenic pseudomonads through the extensive application of MLSA and by employing ANI analysis.

We clarified the taxonomic position of a group of strains that are genomically very closely related to each other, constructing a tight and coherent phylogenetic cluster showed ANI values higher than 97.5% and high TETRA scores in all reciprocal comparisons; therefore these strains should belong to the same species. The presence of more than one single and well-characterized biological entity within the species revealed the existence of a complex and phytopathologically diversified species. In fact, we found that *P. avellanae*, BPIC631 (type-strain), the causal agent of hazelnut bacterial canker in Greece, *P. syringae* pv. *theae* NCPPB2598, (type-strain) the causal agent of bacterial tea shoot blight in Japan and *P. syringae* pv. *actinidiae* (type-strain and other three strains), the causal agents of *Actinidia* spp. bacterial canker worldwide, are included in the boundary of the species. According to a DDH study performed by Gardan et al. on *P. syringae* pathovars and on a subsequent molecular and genomic typing, these phytopathogens all belong to genomospecies 8 [5,6,43]. Of note one *P.* s. pv. *morsprunorum* strain, namely M302280, included in our analysis, showed ANI and TETRA values and phylogenetic grouping that consistently suggested its inclusion within the species *P. avellanae*. However, this pathovar shows an evident genetic variability and two races of the pathogen (race 1 and 2) are clearly distinguishable by means of classical biochemical tests and repetitive-sequence PCR and MLSA [44,45,46]. Therefore, the possibility that these two races represent two distinct pathogens cannot be ruled out. The DDH study of Gardan et al. [5] placed two *P.* s. pv. *morsprunorum* strains, namely CFBP2116 (race 1) and NCPPB2995 (race 2 and the pathotype strain of the pathovar), into genomospecies 2 and 3, respectively. Unfortunately, we do not know whether M302280 corresponds to one of these strains. In addition, the transfer of *P.* s. pv. *morsprunorum* as a whole into *P. avellanae* requires further studies using a larger number of representative strains.

Our study also clearly noted that one *Pseudomonas* strain, isolated from a hazelnut tree showing bacterial canker disease in central Italy, which was originally identified as *P. syringae* pv. *avellanae* [3], and later reclassified as *P. avellanae* [4] does not belong to the species *P. avellanae* species as outlined in this paper but rather to the genomospecie 1 sensu Gardan et al. [5] of the *P. syringae* species complex. In fact, genome analyses indicated that this strain is phylogenetically strictly related to other *P.* s. pv. *syringae* strains, with ANI values higher than 95% resulting from the comparison with *P. syringae* pvs syringae B728a, *aceris* M302273 and *pisi* 704B. Thus, our study also partly reconciles previous investigations based on MLSA that actually treated the two *P. avellanae* lineages as belonging to separate phylogroups of the *P. syringae* species complex [11]. Based on the present study, strains of the phylogroup 1 sensu Wang et al. [11] should now be retained within *P. avellanae*, whereas CRAPAV013=ISPaVe013 (i.e., phylogroup 2) and, most probably the related strains isolated from hazelnut trees in central Italy, are genuine *P. syringae* pv. *avellanae* strains. It should be stressed that in central Italy have been repeatedly isolated, from hazelnut trees showing symptoms of bacterial canker, both *P. avellanae* and *P.* s. pv. *avellanae* as revealed in the present study (i.e., strain CRAFRUEC1) and in previous paper [11].

The phylogenetic analysis performed using with wide-genome data of 1,920 proteins and using ML and a Bayesian approaches with the assessment of both concatenated nucleotide and amino acid sequences belonging to seven housekeeping genes, were all congruent with the ANI and TETRA analysis and with the discussed results. Additionally, recent studies, based on a single housekeeping gene, namely *rpoD*, or on MLSA of concatenated nucleotide or protein sequences, recently noted the close relationships between *P. avellanae* BPIC631, *P.* s. pv. *theae* NCPPB2598, different strains of *P.* s. pv. *actinidiae*, and *P.* s. pv. *morsprunorum* NCPPB2995 and M302280 [18,20,47].

Within this context, the assessment of type III effector protein repertoires provided interesting perspectives. In fact, we found both a putative core repertoire of 14 effector proteins and unique effectors for each of the four pathovars in the *P. avellanae* cluster. The effector *hopM1*, which is present in all seven strains differentiated the *P.* s. pv. *actinidiae*-*P.* s. pv. *morsprunorum* strains from the *P.* s. pv. *tomato*-*P.* s. pv. *lachrymans* strains due to a putative recombination event occurred in the effector locus [31]. It is tempting to speculate that such an event could be involved in the pathogenic specialization of the strains and their consequential taxonomic separation.

The ANI and TETRA analyses, in strict agreement with the phylogenetic analyses, also indicated the close relationships of the five strains of *P.* s. pv. *tomato* (genomospecies 3), including DC3000, and one strain of *P.* s. pv. *lacrhymans* (genomospecies 2) with the strains of the *P. avellanae* In fact, all these strains showed ANI values very close to 96%. Richter and Rosselló-Móra [24] established that the species boundary for a robust demarcation of bacterial species is 95-96% using ANI values, and the phylogenetic analysis confirmed such very close relationships. In particular, the Bayesian tree built with the concatenated amino acid sequences revealed that *P.* s. pv. *tomato* and *P.* s. pv. *lachrymans* strains are embedded within the strains of the *P. avellanae* species. In this regard, other studies have outlined the close phylogenetic relationships between such strains with the strains of *P. avellanae* species [18,20,47]. A further and relevant confirmation of the strong relationships between members of the *P. avellanae* species and strains of genomospecies 3 and *P.* s. pv. *lachrymans* can be inferred through the comparison of the core/pan genomes of 19 *P. syringae* strains by Baltrus et al. [31]. These authors found that the strains of clade I, containing *P.* s. pv. *actinidiae* 302091, *P.* s. pv. *morsprunorum* 302280, *P.* s. pv. *tomato* DC3000 and T1, and *P.* s. pv. *lachrymans* 106 clustered apart from the other two *P. syringae* clades. In addition, this clade, composed by a few strains, contained the highest number of core genes in comparison to the core genomes of the wo larger clades. Whether *P.* s. pv. *tomato* and *P.* s. pv. *lachrymans* are divergent members of *P. avellanae* or whether they represent a closely related species deserve further evaluation.

In conclusion, this study demonstrated the existence of a distinct cluster of strains that represent the nucleus of the species *P. avellanae*. A nomenclatural revision of this *taxon* should be postponed for the availability of further genomic data that could clarify the position of the strains currently classified as *P.* s. pv. *morsprunorum*. The revision should be also elaborated within the overall context of the nomenclatural revision of the *P. syringae* species complex, to coherently address the issued posed by the strict relationships between the strain clusters. Due to the priority rules according to the International Code of Nomenclatura of Bacteria [48,49], the taxonomic revision of the *P. syringae* species complex may result in the need for name changes that may be confounding and should, therefore, be attempted with caution [50].

## Materials and Methods

### Library preparation and genome sequencing

Bacterial genomic DNA was extracted from 1 ml of overnight *P. avellanae* BPIC631 and CRAFRU EC1, *P.* s. pv. *theae* NCPPB2598, *P.* s. pv. *syringae* CRAFRU11 and CRAFRU12 cultures grown in KB broth DNA using a Wizard DNA purification kit (Promega Italia, Padova, Italy) following the manufacturer’s instructions. The identification of *P. avellanae* CRAFRUEC1 and *P.* s. pv. *syringae* CRAFRU 11 and 12 was achieved using well established techniques [8,51]. DNA was measured and checked for quality using a NanoDrop (NanoDrop products, Wilmington, DE, USA). A total of 10 mg of DNA from each sample was fragmented by incubation for 70 min with 5 ml of dsDNA Fragmentase (New England Biolabs, MA, USA). The reaction was stopped with EDTA and purified using a QIAquick PCR purification kit (QIAGEN, Hilden, Germany). The eluate was end repaired using an End Repair kit (New England Biolabs, MA, USA) for 30 min at 20µC. The end-repaired DNA was Atailed for 30 min at 37µC using a d-A Tailing kit (New England Biolabs, MA, USA). After purification using the MinElute purification kit (QIAGEN), the DNA was ligated using Quick T4 DNA ligase (New England Biolabs) to 500 pmol of Illumina adaptors that had been previously annealed by heating at 98µC for 3 min and then slowly cooling to 16µC in a thermocycler. After further purification using the MinElute purification kit (QIAGEN), 1 ml of each reaction was quantified by labelling with biotin, spotted on nitrocellulose after a serial dilution, and detected using an anti-biotin-AP conjugate (Roche Diagnostics, Monza, Italy) following manufacturer’s instructions. Equal amounts of DNA from samples were pooled together and size fractionated by 2% MS-6 agarose (Conda, Madrid, Spain) gel electrophoresis in TAE buffer at 120 V for 60 min. Gel slices containing DNA in the 400 to 600 bp estimated range were cut and purified using QIAquick gel extraction kit (QIAGEN) and used for sample preparation according to the protocol for genomic DNA sequencing using the Illumina HiSeq2000 (Illumina, USA). The samples were run at the Istituto di Genomica Applicata (Udine, Italy).

### Sequencing, assembly, and annotation

Paired reads were assembled into contigs using the *de novo* (i.e. without using a reference genome) assembly option of the CLC genomic workbench (CLC-bio, Aarhus, Denmark) by setting the default parameters. Contigs sequences were scanned for ORFs by GLIMMER, version 3.02[52]. which had been previously trained on the complete genome sequences of *P.* s. pv. *tomato* DC3000 (NC_004578.1, i.e. *Pto* DC3000), *P.* s. pv. *phaseolicola* 1448A (NC_005773.3, i.e. *Pph* 1448A), and *P.* s. pv. *syringae* B728a (NC_007005.1, i.e. *Psy* B728a). The putative proteins were annotated against the RefSeq database using a PERL script for recursive BLASTX searches. Additional genome sequence analyses was performed with the aid of the software packages MUMmer 3.0 [53] and MAUVE [54]. Several *ad hoc* PERL scripts were developed to assist the comparison of genome sequence drafts and their putative protein complement with respect to *P. avellanae*, *P.* s. pv. *th*eae and *P.* s. pv. *syringae* strains, and *P.* s. pv. *tomato* DC3000, *P.* s. *pv. phaseolicola* 1449A and *P.* s. pv. *syringae* B728a.

### Average Nucleotide Identity (ANI) and tetranucleotide frequency correlation coefficients (TETRA) analysis

The analysis of sequences for the determination of their relatedness according to the Average Nucleotide Identity (ANI) and tetranucleotide frequency correlation coefficients (TETRA) were performed with the software JSpecies [24]. The analysis regarded 27 genomes belonging to seven out of nine genomospecies sensu Gardan et al. [5]. Due to absence of genomes in databank, strains of genomospecies 5 and 7 were not analysed. In addition, *P. fluorescens* A506 and *P* *. putida* UW4 were included into the assessment as outgroups (see also Table 2). ANI was calculated using algorithms obtained with the data structure named suffix tree and the MUMmer algorithm implementation [53]. TETRA was used as an alignment-free genomic similarity index as oligonucleotide frequencies carry a species-specific signal. The use of a tetranucleotide usage pattern has been shown to be a good compromise between signal strength and need computational power [24]. Pairwise comparison between genomes is performed by plotting the corresponding tetranucleotide frequency and then obtaining a regression line A graphic representation of the pair-wise relationships between members of *P. avellanae* species has been obtained by using an R statistic software [55].

### Phylogeny based on MLSA

In order to evaluate the evolutionary relationships of the 27 phytopathogenic *Pseudomonas* spp strains, we built five phylogenetic trees. *P. fluorescens* A506 and *P* *. putida* UW4 strains were used as outgroups. Maximum Likelihood (ML) and the Bayesian method analysis were performed with both nucleotide and amino acids sequences using seven housekeeping genes (*argS, dnaQ, gltA, gyrB, recA, rpoB* and *rpoD*), for a total of 6,579 nt and 2,193 aa, respectively. ML analysis was inferred with with PhyML version 3.0 [56], with 1,000 bootstrap replicates, whereas for the Bayesian method we used MrBayes version 3.2.1 [57] with 100,000 generations. To select the best fit model for ML and Bayesian trees, we used the jModelTest [58] and ProtTest [59]. GTR + I + G and Dayoff + G + F were used as best substitution models for nucleotide and amino acids, respectively. The four trees were visualized using FigTree software, version 1.1.2 (http://tree.bio.ed.ac.uk/software/figtree/). In addition, a split network tree, based on concatenation of 6,579 nucleotides from 27 phytopathogenic pseudomonads and *P. fluorescens* A506 and *P* *. putida* UW4 strains as outgroups, was built using the neighbor-joining (NJ) algorithm with the Hamming distance method, obtained using the Splits-Tree software [27]. Bootstrap analysis with 1,000 replications was performed by using the same software.

### Genome wide phylogeny

A data set containing ortholog alignment was prepared using a multistep procedure based on several *ad hoc* Perl scripts. First, the predicted protein sequences of all genomes were analyzed for the identification superfamilies of homologs by a procedure based on reciprocal smallest distance algorithm [60]. Subsequent application of the branch clustering algorithm BranchClust [61], allowed delineation of families of orthologs within superfamilies containing one or more paralogous gene families. The families were then selected, excluding those that did not comprise one protein per each genome or that contained more than one protein for at least one genome, those that did not pass a quality check (i.e. with a mean < 0.7 or a standard deviation < 0.05 in the identity values calculated between all pairs of proteins) and those that contained at least one sequence comprising more than 4% of the positions as internal indels. In total, 1,920 protein sequence alignments, spanning 612,057 amino acid sites, were selected for phylogenetic analysis. Such criteria were also used to point out the core and pan genome of *P. avellanae* species. The trees from each individual DNA sequence alignments were obtained by recursively running PhyML [56] using LC as a substitution model and Nearest Neighbor Interchange (NNI) for tree topology estimate. From the 1,920 protein sequence alignment ML trees, a consensus network was obtained with SplitTree4, using a mean network construction [27]. These networks display edges that occur in a proportion of the gene trees higher than a threshold value. Thus, the presence of reticulation in the network indicates contradictory evidence for grouping [28].

The putatively coded protein sequences were also concatenated to obtain a single large alignment that was submitted to ML analysis with PhyML [56] using LC as a substitution model. Tree topologies were estimated using the better topology obtained using Nearest Neighbor Interchange (NNI) or Subtree Pruning and Regrafting (SPR). The support of the data for each internal branch of the phylogeny was estimated using non-parametric bootstrap with 100 replicated. The number of polymorphic sites analyzed was 59,308. Concatenated gene sequence data were also analyzed using split networks with the aid of the software SplitTree4 [27]. Split networks are used to represent incompatible and ambiguous signals in a data set. The split network used here (i.e., NeighborNet) [62] was computed from ML protein distance estimates using an equal angle algorithm [63] and is depicted as a tree with additional edges, so that the distance between two *taxa* is equal to the length of the shortest path connecting them. It is therefore capable of highlighting *taxa* relationships that are not tree-like.

### Core and pan genome of *P. avellanae* species

The data set containing ortholog alignments, prepared as described above in the section “genome wide phylogenetic analysis”, was used for the selection of shared versus specific genes among the seven strains here described as *P. avellanae*. Concerning the pan genome, for the ORFs assembly we have selected the families as obtained from the genome wide analysis and additionally showing > 60% length hit.

## Supporting Information

Figure S1 **Bayesian phylogenetic tree showing relationships among representative strains of phytopathogenic *Pseudomonas* species and *P. syringae* pathovars.** The phylogenetic tree was constructed using 6,579 concatenated nucleotides of seven housekeeping genes (*argS*, dnaQ*, gltA, gyrB*, recA*, rpoB* and *rpoD*) with bootstrap values (100,000 generations) shown at the nodes. Strain members of genomospecies 8 (*P. avellanae*) sensu Gardan et al. [5], including also *P*. *s*. pv. *morsprunorum* M302280, are shown in red, whereas strain members of genomospecies 2 (P. *s*. pv. *lachrymans* M302278) and 3 (P. *s*. pv. tomato) are in blue. *P. fluorescens* A506 and *P* *. putida* UW4 were included as outgroups. The interior node values of the tree are clade credibility values based on the posterior credibility values produced by MrBayes.
(TIF)

Figure S2 **Phylogenetic relationships among representative strains of phytopathogenic *Pseudomonas* species and *P. syringae* pathovars.** The phylogenetic tree was constructed using 2,193 concatenated amino acid sequences with bootstrap values greater than 65 per cent (1000 replicates) shown at the nodes. The phylogenetic relationships were inferred using the maximum likelihood (ML) method and the Dayoff + G + F as the best model with the PHYLIP package. Strain members of genomospecies 8 (*P. avellanae*) sensu Gardan et al. [5], including also *P. s*. pv. *morsprunorum* M302280, are shown in red, whereas strain members of genomospecies 2 (*P. s*. pv. *lachrymans* M302278) and 3 (*P. s*. pv. *tomato*) are in blue. *P. fluorescens* A506 and *P* *. putida* UW4 were included as outgroups.
(TIF)

Table S1 **Average nucleotide identity (ANI) values calculated between genomes of 20 representative *Pseudomonas* strains belonging to genomospecies 1, 2, 3, 4, 6, 8 and 9 sensu Gardan et al. [5], and *P. fluorescens* A506 and *P* *. putida* UW4 as outgroups.** Values higher than 95% are in boldface. The ANI values of *P. avellanae* species strains and *P. s*. pv. *tomato* DC3000 and *P. s*. pv. *lachrymans* M32278 are also pointed out in grey.
(DOCX)
