# Supplementary figures and images for: A Genomic Redefinition of Pseudomonas avellanae species
Source: PLoS One. 2013 Sep 25;8(9):e75794. doi: 10.1371/journal.pone.0075794 (PMC3783423; doi:10.1371/journal.pone.0075794)

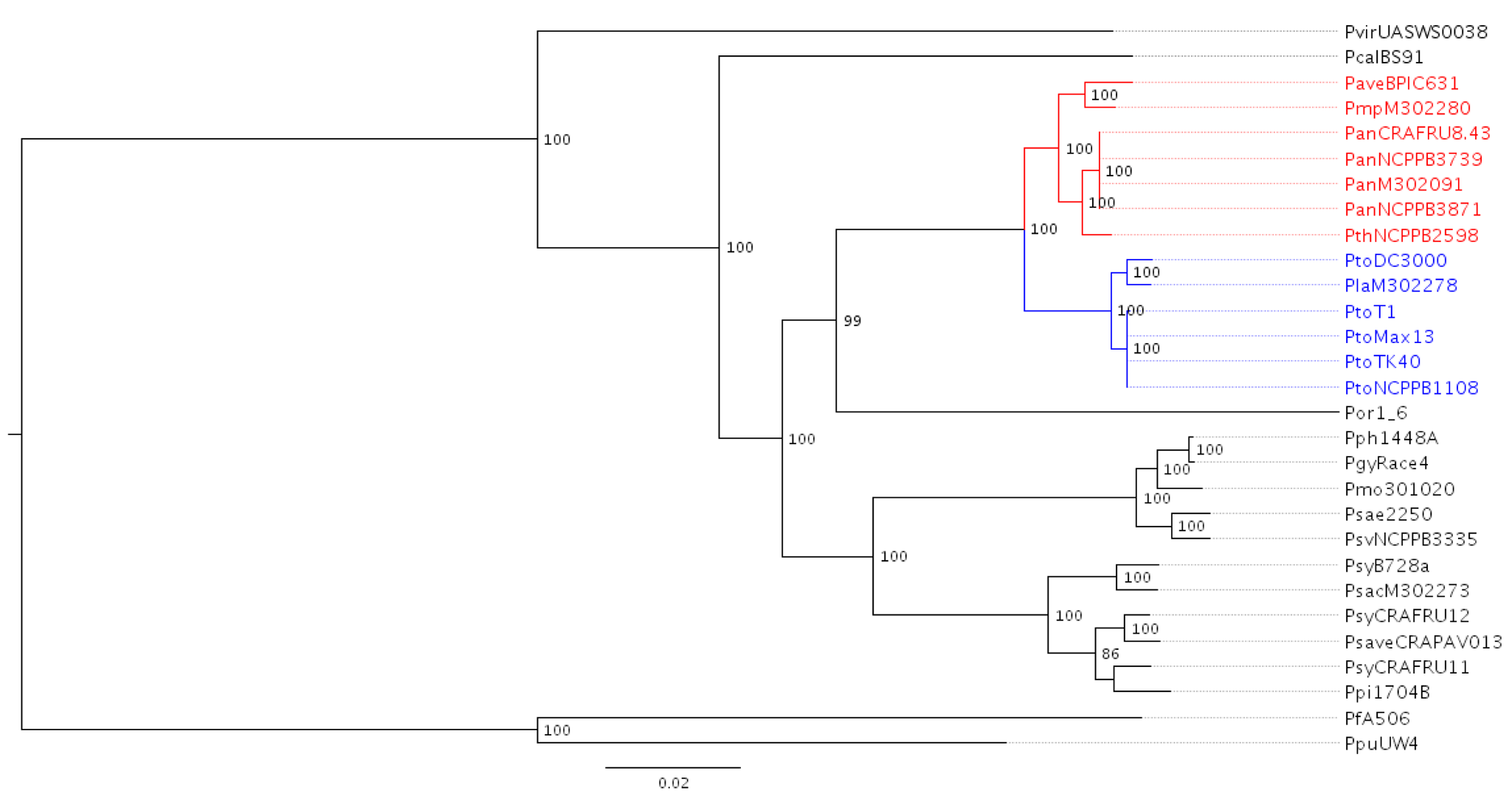

Supplement: Figure S1 — Bayesian phylogenetic tree showing relationships among representative strains of phytopathogenic Pseudomonas species and P. syringae pathovars. The phylogenetic tree was constructed using 6,579 concatenated nucleotides of seven housekeeping genes (argS, dnaQ, gltA, gyrB, recA, rpoB and rpoD) with bootstrap values (100,000 generations) shown at the nodes. Strain members of genomospecies 8 (P. avellanae) sensu Gardan et al. [5], including also P. s. pv. morsprunorum M302280, are shown in red, whereas strain members of genomospecies 2 (P. s. pv. lachrymans M302278) and 3 (P. s. pv. tomato) are in blue. P. fluorescens A506 and P . putida UW4 were included as outgroups. The interior node values of the tree are clade credibility values based on the posterior credibility values produced by MrBayes. (TIF) [file pone.0075794.s001.tif]

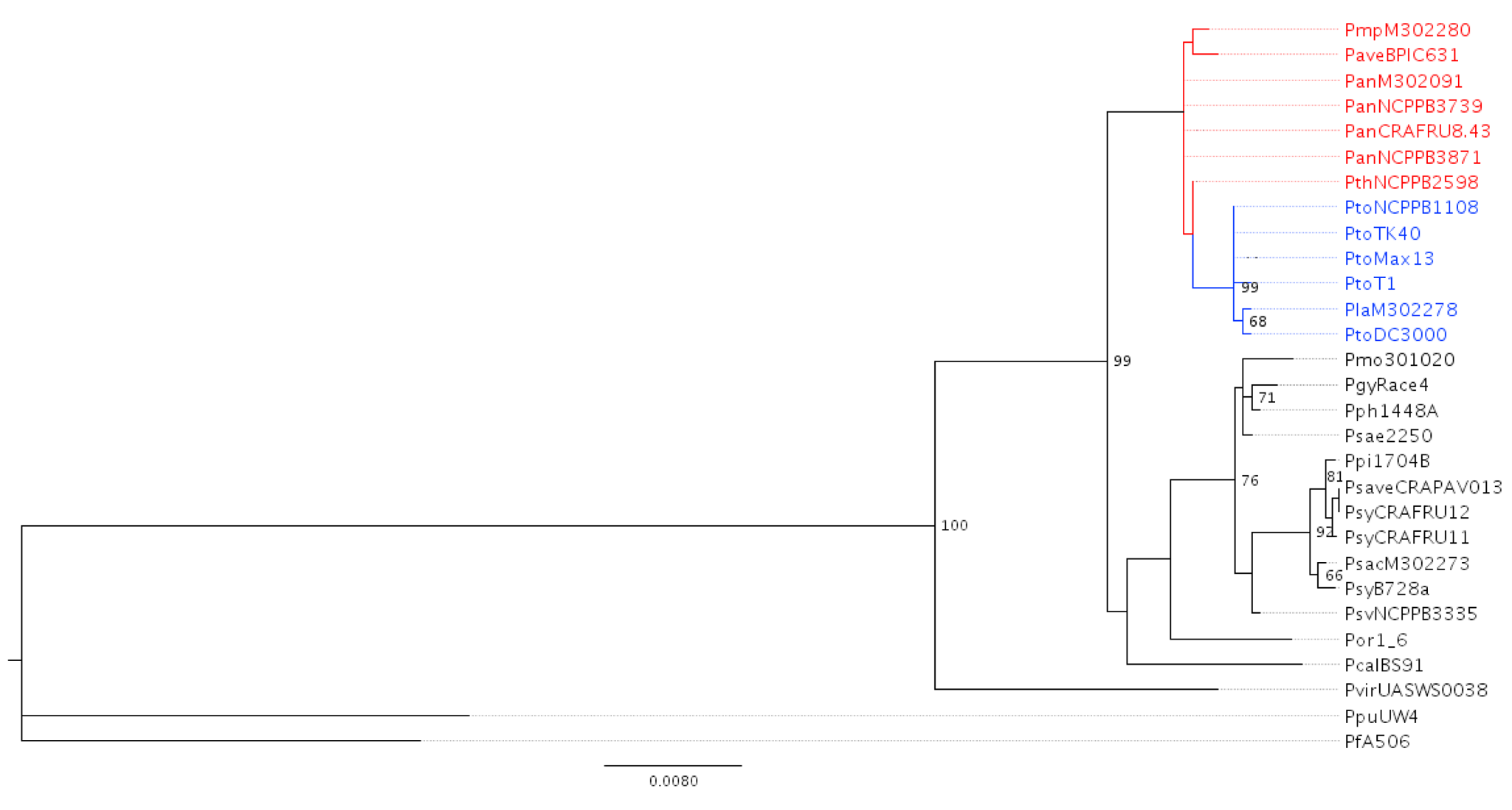

Supplement: Figure S2 — Phylogenetic relationships among representative strains of phytopathogenic Pseudomonas species and P. syringae pathovars. The phylogenetic tree was constructed using 2,193 concatenated amino acid sequences with bootstrap values greater than 65 per cent (1000 replicates) shown at the nodes. The phylogenetic relationships were inferred using the maximum likelihood (ML) method and the Dayoff + G + F as the best model with the PHYLIP package. Strain members of genomospecies 8 (P. avellanae) sensu Gardan et al. [5], including also P. s. pv. morsprunorum M302280, are shown in red, whereas strain members of genomospecies 2 (P. s. pv. lachrymans M302278) and 3 (P. s. pv. tomato) are in blue. P. fluorescens A506 and P . putida UW4 were included as outgroups. (TIF) [file pone.0075794.s002.tif]
