# Supplementary material for: A Genomic Redefinition of Pseudomonas avellanae species
Source: PLoS One. 2013 Sep 25;8(9):e75794. doi: 10.1371/journal.pone.0075794 (PMC3783423; doi:10.1371/journal.pone.0075794)
Supplement: Table S1 — Average nucleotide identity (ANI) values calculated between genomes of 20 representative Pseudomonas strains belonging to genomospecies 1, 2, 3, 4, 6, 8 and 9 sensu Gardan et al. [5], and P. fluorescens A506 and P . putida UW4 as outgroups. Values higher than 95% are in boldface. The ANI values of P. avellanae species strains and P. s. pv. tomato DC3000 and P. s. pv. lachrymans M32278 are also pointed out in grey. (DOCX) [file pone.0075794.s003.docx]

Table S1. Average nucleotide identity (ANI) values calculated between genomes of 20 representative *Pseudomonas* strains belonging to genomospecies 1, 2, 4, 6, 8 and 9 *sensu* Gardan et al. [5], and *P. fluorescens* A506 and *P. putida* UW4 as outgroups. Values higher than 95% are in boldface. The ANI values of strains of *P. avellanae* are also pointed out in grey.

| ANIm | Gen.1 | | | | Gen.2 | | | | | | Gen.3 | | Gen.4 | Gen.6 | Gen.8 | | | Gen.9 |  |  |
| --- | --- | --- | --- | --- | --- | --- | --- | --- | --- | --- | --- | --- | --- | --- | --- | --- | --- | --- | --- | --- |
|  | PsyB728a | PsacM302273 | Ppi1704B | PsaveCRAPAV013 | Psae2250 | PgyRace4 | Pmo301020 | Pph1448A | PsvNCPPB3335 | PlaM302278 | PtoDC3000 | PmpM302280 | Por1_6 | PvirUASWS0038 | PaveBPIC631 | PanNCPPB3739 | PthNCPPB2598 | PcalBS91 | PfA506 | PputUW4 |
| PsyB728a | --- | **98,60** | **95,37** | **95,54** | 89,75 | 89,95 | 89,83 | 89,69 | 89,75 | 87,92 | 87,97 | 87,99 | 87,48 | 86,10 | 87,86 | 87,93 | 87,86 | 87,43 | 84,47 | 84,69 |
| PsacM302273 | **98,61** | --- | **95,33** | **95,53** | 89,79 | 90,00 | 89,82 | 89,73 | 89,80 | 87,99 | 87,93 | 88,03 | 87,38 | 86,19 | 87,90 | 88,01 | 87,94 | 87,51 | 84,54 | 84,81 |
| Ppi1704B | **95,39** | **95,35** | --- | **96,68** | 90,08 | 90,30 | 90,15 | 89,95 | 90,06 | 88,49 | 88,54 | 88,52 | 87,86 | 86,60 | 88,47 | 88,26 | 88,27 | 88,07 | 84,86 | 85,21 |
| PsaveCRAPAV013 | **95,54** | **95,53** | **96,66** | --- | 89,78 | 89,89 | 89,80 | 89,62 | 89,75 | 87,92 | 87,93 | 87,91 | 87,47 | 86,09 | 87,81 | 87,88 | 87,79 | 87,42 | 84,50 | 84,77 |
| Psae2250 | 89,75 | 89,79 | 90,10 | 89,78 | --- | **97,85** | **97,84** | **97,80** | **98,55** | 88,37 | 88,48 | 88,58 | 87,28 | 85,94 | 88,54 | 88,52 | 88,49 | 87,56 | 84,52 | 84,70 |
| PgyRace4 | 89,93 | 89,98 | 90,30 | 89,88 | **97,88** | --- | **98,20** | **99,45** | **97,84** | 88,68 | 88,74 | 88,84 | 87,58 | 86,33 | 88,92 | 88,79 | 88,87 | 87,92 | 84,82 | 84,92 |
| Pmo301020 | 89,82 | 89,81 | 90,16 | 89,80 | **97,88** | **98,21** | --- | **98,20** | **97,84** | 88,51 | 88,58 | 88,71 | 87,42 | 86,14 | 88,76 | 88,74 | 88,65 | 87,76 | 84,80 | 84,89 |
| Pph1448A | 89,68 | 89,73 | 89,96 | 89,62 | **97,81** | **99,42** | **98,17** | --- | **97,75** | 88,25 | 88,27 | 88,47 | 87,19 | 85,85 | 88,46 | 88,54 | 88,38 | 87,29 | 84,41 | 84,55 |
| PsvNCPPB3335 | 89,74 | 89,80 | 90,08 | 89,75 | **98,54** | **97,81** | **97,81** | **97,74** | --- | 88,34 | 88,34 | 88,52 | 87,23 | 85,96 | 88,53 | 88,44 | 88,51 | 87,45 | 84,58 | 84,66 |
| PlaM302278 | 87,93 | 87,99 | 88,52 | 87,92 | 88,36 | 88,71 | 88,53 | 88,26 | 88,35 | --- | **99,28** | **95,87** | 87,83 | 86,06 | **95,91** | **95,77** | **95,79** |  |  |  |
| PtoDC3000 | 87,97 | 87,93 | 88,53 | 87,93 | 88,44 | 88,73 | 88,56 | 88,25 | 88,32 | **99,29** | --- | **95,84** | 88,09 | 86,12 | **95,86** | **95,68** | **95,76** | 88,75 | 84,37 | 84,47 |
| PmpM302280 | 87,99 | 88,04 | 88,54 | 87,92 | 88,58 | 88,87 | 88,74 | 88,48 | 88,53 | **95,87** | **95,84** | --- | 87,82 | 86,09 | **97,83** | **98,02** | **98,05** | 88,58 | 84,46 | 84,60 |
| Por1_6 | 87,58 | 87,43 | 88,00 | 87,53 | 87,31 | 87,61 | 87,44 | 87,20 | 87,24 | 87,88 | 88,13 | 87,84 | --- | 85,97 | 87,79 | 87,76 | 87,75 | 87,85 | 84,55 | 84,64 |
| PvirUASWS0038 | 86,04 | 86,13 | 86,56 | 86,01 | 85,88 | 86,29 | 86,09 | 85,84 | 85,91 | 85,99 | 86,00 | 86,02 | 85,87 | --- | 86,00 | 86,06 | 86,03 | 86,01 | 84,51 | 84,58 |
| PaveBPIC631 | 87,86 | 87,90 | 88,48 | 87,82 | 88,54 | 88,95 | 88,80 | 88,45 | 88,52 | **95,92** | **95,86** | **97,83** | 87,75 | 86,06 | --- | **97,63** | **97,59** | 88,50 | 84,42 | 84,52 |
| PanNCPPB3739 | 87,93 | 88,01 | 88,30 | 87,88 | 88,52 | 88,80 | 88,77 | 88,55 | 88,45 | **95,78** | **95,69** | **98,04** | 87,70 | 86,13 | **97,67** | --- | **98,95** | 88,40 | 84,46 | 84,67 |
| PthNCPPB2598 | 87,86 | 87,93 | 88,30 | 87,78 | 88,49 | 88,89 | 88,66 | 88,38 | 88,52 | **95,80** | **95,75** | **98,06** | 87,73 | 86,09 | **97,61** | **98,94** | --- | 88,48 | 84,42 | 84,54 |
| PcalBS91 | 87,43 | 87,51 | 88,10 | 87,43 | 87,57 | 87,98 | 87,81 | 87,32 | 87,46 | 88,53 | 88,79 | 88,58 | 87,83 | 86,06 | 88,51 | 88,41 | 88,48 | --- | 84,39 | 84,66 |
| PfA506 | 84,46 | 84,53 | 84,87 | 84,48 | 84,50 | 84,81 | 84,79 | 84,40 | 84,56 | 84,40 | 84,36 | 84,45 | 84,47 | 84,79 | 84,41 | 84,44 | 84,41 | 84,39 | --- | 85,93 |
| PpuUW4 | 84,70 | 84,81 | 85,22 | 84,76 | 84,70 | 84,93 | 84,88 | 84,56 | 84,66 | 84,64 | 84,47 | 84,59 | 84,52 | 84,82 | 84,52 | 84,67 | 84,54 | 84,67 | 85,95 | --- |
